# Comparative Studies of the Gut Microbiota in the Offspring of Mothers With and Without Gestational Diabetes

**DOI:** 10.3389/fcimb.2020.536282

**Published:** 2020-10-23

**Authors:** Mie Korslund Wiinblad Crusell, Tue Haldor Hansen, Trine Nielsen, Kristine Højgaard Allin, Malte C. Rühlemann, Peter Damm, Henrik Vestergaard, Christina Rørbye, Niklas Rye Jørgensen, Ole Bjarne Christiansen, Femke-Anouska Heinsen, Andre Franke, Torben Hansen, Jeannet Lauenborg, Oluf Pedersen

**Affiliations:** ^1^Novo Nordisk Foundation Center for Basic Metabolic Research, Section for Metabolic Genetics, University of Copenhagen, Copenhagen, Denmark; ^2^Department of Obstetrics and Gynaecology, Hvidovre University Hospital, Hvidovre, Denmark; ^3^Department of Clinical Epidemiology, Bispebjerg and Frederiksberg Hospital, Copenhagen, Denmark; ^4^Institute of Clinical Molecular Biology, Christian-Albrechts University, Kiel, Germany; ^5^Center for Pregnant Women With Diabetes, Department of Obstetrics, Rigshospitalet University Hospital, Copenhagen, Denmark; ^6^Department of Clinical Medicine, Faculty of Health and Medical Sciences, University of Copenhagen, Copenhagen, Denmark; ^7^Department of Clinical Biochemistry, Rigshospitalet University Hospital, Copenhagen, Denmark; ^8^OPEN, Odense Patient Data Explorative Network, Odense University Hospital/Institute of Clinical Research, University of Southern Denmark, Odense, Denmark; ^9^Department of Obstetrics and Gynaecology, Rigshospitalet University Hospital, Copenhagen, Denmark; ^10^Fertility Clinic, Rigshospitalet University Hospital, Copenhagen, Denmark; ^11^Department of Obstetrics and Gynaecology, Aalborg University Hospital, Aalborg, Denmark; ^12^Department of Obstetrics and Gynaecology, Herlev University Hospital, Herlev, Denmark

**Keywords:** gut microbiota, infancy, gestational diabetes, maternal glycaemic traits, bacterial genera

## Abstract

**Background:** Offspring of mothers with gestational diabetes mellitus (GDM) have increased risk of developing metabolic disorders as they grow up. Microbial colonization of the newborn gut and environmental exposures affecting the configuration of the gut microbiota during infancy have been linked to increased risk of developing disease during childhood and adulthood. In a convenience sample, we examined whether the intestinal tract of children born to mothers with GDM is differentially colonized in early life compared to offspring of mothers with normal gestational glucose regulation. Secondly, we examined whether any such difference persists during infancy, thus potentially conferring increased risk of developing metabolic disease later in life.

**Methods:** Fecal samples were collected from children of mothers with (*n* = 43) and without GDM (*n* = 82) during the first week of life and again at an average age of 9 months. The gut microbiota was characterized by 16S rRNA gene amplicon sequencing (V1–V2). Differences in diversity and composition according to maternal GDM status were assessed, addressing potential confounding by mode of delivery, perinatal antibiotics treatment, feeding and infant sex.

**Results:** Children of mothers with GDM were featured by a differential composition of the gut microbiota, both during the first week of life and at 9 months, at higher taxonomic and OTU levels. Sixteen and 15 OTUs were differentially abundant after correction for multiple testing during the first week of life and at 9 months, respectively. Two OTUs remained differentially abundant after adjustment for potential confounders both during the first week of life and at 9 months. Richness (OTU) was decreased in neonates born to mothers with GDM; however, at 9 months no difference in richness was observed. There was no difference in Shannon's diversity or Pielou's evenness at any timepoint. Longitudinally, we detected differential changes in the gut microbiota composition from birth to infancy according to GDM status.

**Conclusion:** Differences in glycaemic regulation in late pregnancy is linked with relatively modest variation in the gut microbiota composition of the offspring during the first week of life and 9 months after birth.

## Introduction

Events occurring in early life, even prenatally, are known to affect the risk of developing childhood and adult disease (Catalano and deMouzon, 2015; Wallace et al., 2016). The Barker hypothesis—or “developmental origins of adult disease” hypothesis—states that adverse influences during fetal life may result in permanent physiological and metabolic alterations conferring an increased disease risk in adulthood (Barker, 2007). Pre- and perinatal priming of the fetal and neonatal gut microbiota could be one of many mechanisms underlying the increased risk of disease.

The uterine environment was until recently considered sterile and the first microbial colonization of the gastrointestinal tract was believed to occur during the foetus' passage through the birth canal. However, recent studies have shown that human meconium contains bacterial DNA and specific bacteria fed to pregnant mice are detectable in the meconium of the offspring, suggesting that colonization of the fetal gastrointestinal tract may occur intrauterine and is directly influenced by maternal environmental exposures (Jiménez et al., 2008; Hu et al., 2013). Different exposures during and after birth, such as feeding patterns, mode of delivery, and antibiotics treatment are known to impact the composition of the gut microbiota during the first year of life (Palmer et al., 2007; Bergström et al., 2014; Bäckhed et al., 2015; Azad et al., 2016; Martin et al., 2016; Rutayisire et al., 2016).

One of the most common medical disorders in pregnancy is gestational diabetes mellitus (GDM) which is characterized by insufficient secretion of insulin in response to increased insulin resistance resulting in hyperglycaemia (Lauenborg et al., 2004; Buckley et al., 2012). Maternal hyperglycaemia is associated with increased fetal growth and perinatal morbidity (The HAPO Study Cooperate Research Group, 2008) and longitudinal studies have shown that offspring of women with GDM have increased risk of becoming overweight and developing the metabolic syndrome, and pre-diabetes or overt diabetes (Clausen et al., 2008, 2009; Nehring et al., 2013; Damm et al., 2016). Similarly, overt type 2 diabetes in pregnant women is linked with altered bacterial composition of the meconium of their infants (Hu et al., 2013).

We tested the hypotheses (a) that the gastrointestinal tract of neonates of mothers with GDM is differentially colonized during first week of life compared with neonates born to mothers with normal gestational glucose regulation, and (b) that if any differences exist, they persist during infancy, thus potentially contributing to the pathophysiological processes leading to development of metabolic diseases later in life.

## Methods and Materials

### Study Population and Design

The data presented in the present manuscript are derived from a convenience sample of mothers with GDM and their offspring (Crusell et al., 2018). The study sample included 125 offspring of mothers with or without GDM. Normal glucose regulation or GDM was determined from of an 75 g 2 h (2-h) oral glucose tolerant test (OGTT) performed in pregnant women of third trimester according to the International Association of the Diabetes and Pregnancy Study Group criteria for GDM diagnosis (International Association of Diabetes Pregnancy Study Groups, 2010), resulting in 43 offspring born to mothers with GDM and 82 offspring born to mothers with normal gestational plasma glucose regulation, hence forward referred to as “mothers without GDM”. Preterm birth was defined as birth before 37+0 gestational week according to WHO (World Health Organization, 2015).

### Inclusion Criteria for Pregnant Women

**Figure 1:**
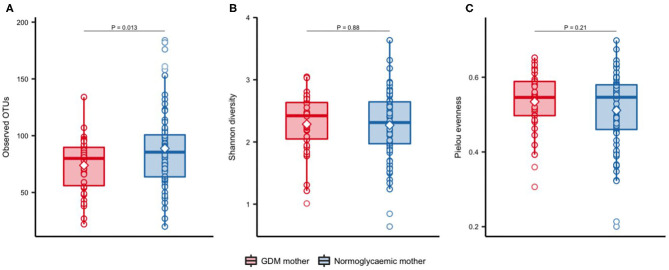
Alpha-diversity of gut microbiota in newborns of mothers with GDM and normoglycaemic mothers. Observed OTUs **(A)**, Shannon's diversity **(B)**, and Pielou's evenness **(C)**, in newborns of mothers with GDM (*n* = 34) and of normoglycaemic mothers (*n* = 72). Samples were rarefied to an equal depth of 10,000 reads. Boxes represent interquartile range (IQR), with the inside line representing the median. Circles represent individual samples. Differences between newborn of mothers with GDM and newborns of normoglycaemic mothers were tested using Students *t*-test.

Detailed information and description of the mothers have been reported (Crusell et al., 2018). In brief, the mothers of the offspring in the present cohort had been referred to a 2-h 75 g OGTT in their third trimester (27–33 gestational week) due to the presence of one or more of specific risk factors for GDM including family history of type 2 diabetes mellitus; previous delivery of a child weighing ≥4,500 g at birth; glucosuria; pre-pregnancy BMI ≥27 kg/m^2^ or known polycystic ovarian syndrome (Additional file 2: Supplementry Figure 1). For the present study only women who completed two project visits were included; the first in third trimester of pregnancy and the second at an average of 8.8 months after delivery, resulting in a study sample of 125 women. The women were recruited from two University Hospitals in the Copenhagen Capital Region, Hvidovre and Herlev. None of the participating women had received antibiotic treatment within a period of 2 months before the date for the OGTT test. None of the women received any medical treatment for their GDM at any time point of the study.

### Information Derived From Medical Records and Questionnaires

Information about gestational age at birth, mode of delivery and use of antibiotics during labor and delivery was obtained from hospital records. Information about the weight and length measurements of the offspring was obtained from registrations in the municipal healthcare system. Information on breastfeeding or formula feeding, use of antibiotics, vaccination, maternal smoking during pregnancy and intake of vitamins or medications during pregnancy were obtained from questionnaires. More than 80% in both groups took vitamin supplements during pregnancy in accordance with the prenatal health recommendation in Denmark. All the infants were breastfeed at least during the first 2 weeks of life.

### Anthropometrical and Physiological Variables of Pregnant Women, Maternal Biochemistry, and Definition of GDM

Anthropometrical and physiological variables of the included women in the study have previously been described in details elsewhere (Crusell et al., 2018). Maternal body-mass index (BMI) was calculated by dividing weight in kg by the square of height in meter. Normal weight was defined as BMI <25 kg/m^2^, overweight as BMI ≥25 and <30 kg/m^2^ and obesity as BMI ≥30 kg/m^2^ (World Health Organization, 2016). Maternal gestational hypertension was defined as development of either a systolic blood pressure ≥140 mmHg, a diastolic blood pressure ≥90 mmHg, or both after 20 weeks of gestation (Tranquilli et al., 2014).

Maternal blood samples in both third trimester and after delivery were collected after an overnight fast. During a standard 2-h 75 g OGTT blood was collected in the fasting state, at 30, 60, and 120 min. Homeostasis model assessment of insulin resistance (HOMA-IR) and insulinogenic index and disposition index were calculated according to Matsuda et al. (mmatsuda.diabetes-smc.jp/MIndex.html) (Matsuda and DeFronzo, 1999). Diagnosis of GDM was according to the International Association of the Diabetes and Pregnancy Study Group (IADPSG) criteria: one or more elevated values: fasting plasma glucose ≥5.1 mmol/L and/or 1-h plasma glucose ≥10.0 mmol/L and/or 2-h plasma glucose ≥8.5 mmol/L (International Association of Diabetes Pregnancy Study Groups, 2010).

### Descriptive Statistical Analyses

Statistical analyses were performed using R Studio version 1.0.136 (http://www.r-project.org/). Two-tailed *t*-test and the chi-square test were used to test for differences in phenotypically characteristics of normal distributed variables. Variables were log-transformed if they were not normally distributed. *P*-values below 0.05 were considered statistically significant.

### Microbiota Analysis

#### Fecal Sampling

Fecal samples (two samples) were collected at home by the parents 2 days after the offspring had passed meconium (within the first week of life, (range 2–7 days of life, *n* = 123) and again at least 6 months after birth [8.8 months on average (8–9 months representing 25 and 75th percentiles, range = 6–18 months, *n* = 124]. After collection, the samples were immediately stored at −18°C and transferred on dry ice to a −80°C storage facility within 48 h until DNA extraction.

#### DNA Extraction, Library Preparation, 16S rRNA Amplicon Gene Sequencing and Quality Assurance

NucleoSpin Soil kit (Macherey-Nagel) was used to extract total fecal genomic DNA from 150 to 200 mg of stool (Bäckhed et al., 2015). The fecal material was suspended in SL2 buffer containing SX enhancer and cell disruption was carried out by bead beating at 30 Hz for 5 min using a TissueLyser instrument (Qiagen). DNA extraction from 17 newborn samples and 1 infant sample failed, leaving 106 newborn samples and 123 infant samples available for amplification and sequencing.

The 16S rRNA gene was amplified using the 27F/338R primer pair targeting the V1–V2 region described in Caporaso et al. (2011). Sequencing was performed on the Illumina MiSeq platform using a dual MID indexing (8-nt barcodes each on forward- and reverse primer) as described by Kozich et al. with MiSeq Reagent Kits v3 (Kozich et al., 2013). Demultiplexing after sequencing was done in CASAVA 1.8.2 allowing no mismatches in the index sequence. The raw sequencing data was trimmed using sickle (Joshi and Fass, 2011) in paired-end mode, using default quality values and a minimum read length of 100 bp after trimming. Forward and reverse reads were merged using vsearch (v1.9, https://github.com/torognes/vsearch), setting minimal and maximal overlap to 280 and 350 bp, respectively. Further quality control included filtering based on a maximum expected error of 1 (vsearch v1.9) and a read quality of below 30 in maximum 5% of the sequences, followed by reference based chimera detection using vsearch. Quality controlled reads were annotated using UTAX (Edgar, 2013) and sequences that could not be assigned to bacteria and sequences assigned to chloroplasts were removed. Picking of Operational Taxonomic Units (OTUs) was performed using vsearch based on the non-rarefied dataset and an identity cut-off of 97%, excluding only singleton sequences prior to clustering. OTU picking was followed by a second step identifying chimeras, this time using a *de novo* approach. The reads of each sample were mapped to these OTU sequences and 10,000 randomly chosen sequences were used to construct the OTU abundance table. Additionally, OTU sequences underwent taxonomic annotation using the RDP classifier (Wang et al., 2007) and the training set 16 provided on the RDP website (https://rdp.cme.msu.edu/). The cut-off for annotation was set to a classification score of 0.8, assigning each OTU to the lowest taxonomic level exceeding this threshold in the classification. This classification was used to construct a final taxonomic abundance table based on the OTU abundance table, collapsing OTUs assigned to the same taxon into one taxonomic bin.

#### Alpha Diversity

Alpha diversity metrics (richness and Shannon's index) were calculated using the *phyloseq* R package (Love et al., 2014) based on rarefied OTU counts (10,000 reads per sample). Pielou's evenness index was calculated as Shannon's index/log_e_(richness).

Cross-sectional differences in alpha diversity between offspring of mothers with GDM and offspring of normoglycaemic mothers were assessed using a Student's *T*-test. Confounding effects of maternal pre-pregnancy BMI (continuous variable), offspring sex (categorical), perinatal antibiotic exposure (categorical; exposed vs. unexposed), feeding during infancy (categorical; supplementary formula and currently partially breast fed vs. No formula and currently partially breast fed vs. supplementary formula and currently solids only vs. No formula and currently solids only; adjusted for at infancy only) and mode of delivery (categorical; vaginal vs. C section) were individually adjusted for by linear regression (Diversity ~ GDM status + Covariate). Diversity metrics were mean centered and scaled prior to analyses.

Change in alpha diversity between first week of life and 9 months was assessed using mixed linear regression [R package *lme4* (Bates et al., 2014)] with a random effect of subject. To test for differential change in alpha diversity metrics between time points in offspring of mothers with and without GDM, an ANOVA model was fitted with a two-way interaction between GDM status and time. The independent variable was tested using a *post hoc t*-test. Models were fitted using restricted maximum likelihood, and assumptions were checked visually by inspection of residual plots and normal probability plots. Longitudinal analyses were performed with and without adjustment for pre-pregnancy BMI, mode of delivery, offspring sex, perinatal antibiotics exposure, and feeding during infancy.

Association between alpha diversity metrics and glycaemic traits (fasting and 2 h plasma glucose, insulin sensitivity, and disposition index) was tested using linear regression (Glycaemic traits ~ Diversity). To adjust for confounding effects of maternal pre-pregnancy BMI, offspring sex, perinatal antibiotics exposure, and mode of delivery multifactor adjusted regression test was used (Glycaemic traits ~ Diversity + Covariate). The interaction between maternal GDM status and diversity (Glycaemic traits ~ Diversity + GDM status + Diversity:GDM status) was used to test whether the association between alpha diversity and glycaemic traits differed according to GDM status. This test was adjusted for confounding effects, as mentioned above, on the interaction between maternal GDM status and for diversity a model with all main effects and two-way interactions between independent variables were specified.

#### Beta Diversity

Analyses of community structure were performed using the *vegan* R packages (Oksanen et al., 2012). Cross-sectional difference in community structure between children born to mothers with and without GDM, was assessed by permutational analysis of variance (PERMANOVA) of weighted and un-weighted UniFrac distances, as implemented in the *adonis* function (Distances ~ Covariate + GDM status). Individual confounding effects were adjusted for by including a main effect of each covariate separately (Distances ~ Covariate + GDM status).

For longitudinal analyses of community structure, PERMANOVA models were fitted with permutations constrained within each individual (Distances ~ Time, strata = Subject).

Differential change in community structure between first week of life and 9 months of age in children born to mothers with and without GDM was assessed by contrasting the levels of the interaction between GDM status and time (PERMANOVA model specified as (Distances ~ Time:Status, strata = Subject), where Time:Status is a two-way interaction between GDM status and time, with contrasts specified as GDM:Infant – GDM:Newborn – NGR:Infant + NGR:Newborn). Individual confounding effects were adjusted for by including a main effect of each covariate separately (Distances ~ Covariate + Time×GDM status).

Principal coordinate ordination was performed using the *capscale* function, specifying an unconstrained model (Distances ~ 1). To adjust ordination results for confounding effects each covariate was partialled out separately [Distances ~ 1 + Condition(Covariate)].

#### Composition

To assess cross-sectional differences at OTU level, we performed differential abundance analyses on unrarefied, untransformed OTU tables using a negative binomial Wald test as implemented in the DESeq2 R package (Love et al., 2014). Only OTUs present in at least 20% of individuals regardless of time point (406 of 2,982 OTUs) were considered. OTUs exhibiting differential change between time points in infants born to mothers with and without GDM were identified by a linear mixed model ANOVA, as described for alpha diversity metrics. For the longitudinal analyses rarefied (10,000 sequences per sample) OTU abundances were added a pseudo count equal to the lowest non-zero abundance of each OTU and log transformed. Only results significant at a 10% false discovery rate (*ad modum* Benjamini-Hochberg) are reported.

At deeper taxonomic levels (biomarkers from phylum to genus level) we performed linear discriminant analysis (LDA) using LEfSe (Segata et al., 2011) with default parameters (alpha value for Wilcoxon tests was set at 0.05, the logarithmic LDA score threshold set at 2.0) to identify taxonomic biomarkers that characterize the differences between offspring of mothers with and without GDM.

To test the association of individual OTUs with maternal glycaemic traits during pregnancy, a negative binomial Wald test as implemented in the DESeq2 package was applied. The log2 fold difference in abundance between the upper and lower maternal glycaemic trait tertile was tested using a *post hoc* Wald test with and without adjustment for pre-pregnancy BMI.

## Results

### Physiological Cohort Descriptives

Fecal samples from 43 offspring of mothers with GDM and 82 offspring of mothers without GDM were obtained within the first week of life, hence forward referred to as neonates and first week of life samples, and again at an average of 8.8 months of age, hence forward referred to as infants and in infancy samples. Gestational age, weight and length at birth, mode of delivery, and perinatal antibiotics treatment did not differ between the two groups. Sixteen infants had received antibiotic treatments before 9 months of age. We do not have information on the type of perinatal antibiotic used but the general praxis of antibiotic use in Denmark is Benzylpenicillin (Table 1; Additional file 1: Supplementary Table 1). One fourth of the children (25.6%) were born to mothers who received antibiotics during labor and delivery. Two children born to mothers with GDM and three children born to mothers without GDM were born preterm (at gestational age 30+2, 36+6, and 34+5, 35+5, 35+1). Repeating the analyses without the five preterm children did not significantly change the results. During pregnancy only two women were smoking <7 cigarettes a day. At 9 months after delivery 17 women were smoking, all reporting smoking <10 cigarettes daily. There were no difference between the prevalence of smoking in the two groups in infancy and therefore we did not adjust for this factor in the following analyses. None of the women with GDM were prescribed medication during pregnancy. Only nine of the women had GDM according to national Danish criteria and therefore the majority of the GDM women did not receive intervention during pregnancy.

**Table 1:** Descriptive information about the offspring.

	**Children born to mothers with GDM (*n* = 43)**	**Children born to mothers with normal gestational glucose regulation (*n* = 82)**	***P***
**CHARACTERISTICS AND MEASUREMENTS AT FIRST WEEK OF LIFE**			
Birth weight (g)	3559 (535.6)	3690 (402.0)	0.2
Birth length (cm)	51.8 (2.0)	52.4 (1.9)	0.1
Gestational age at birth (weeks)	39.4 (1.5)	39.7 (1.7)	0.4
Mode of delivery (*n*)	34 (79.1%) vaginal 9 (20.9%) cesarean section	71 (86.6%) vaginal 11 (13.4%) cesarean section	0.4
Antibiotics during labor and delivery (*n*)	12 (27.9%)	20 (24.4%)	0.8
Preterm birth (<= 37 gestational week) (*n*)	2 (4.7%)	3 (3.7%)	1
Sex of infant	22 boys (51.2%) 21 girls (48.8%)	42 boys (51.2%) 40 girls (48.8%)	1
**CHILDREN CHARACTERISTICS FROM BIRTH UNTIL PROJECT VISIT AT 9 MONTHS OF LIFE**			
Children's age in infancy at collection of infant fecal sample (days)	293 (72.6)	274 (54.9)	0.1
Exclusively breastfeeding from birth in months	4.5 (2.1)	4.21 (2.1)	0.5
Breastfeeding at 9 months of life	23 (53.5%)	40 (51.3%)	1

*Data presented as mean (SD) or as n and %. P calculated by two-tailed t-test for continues variables. For categorical variables P is calculated by either chi-square test of fisher's exact test. GA, gestational age. Children age in days at time for collection of infant fecal samples is calculated from birth to the date the mothers participated in the project, filled out questionnaires and second fecal sample*.

### Lower OTU Richness in Neonates of Mothers With GDM

**Figure 2:**
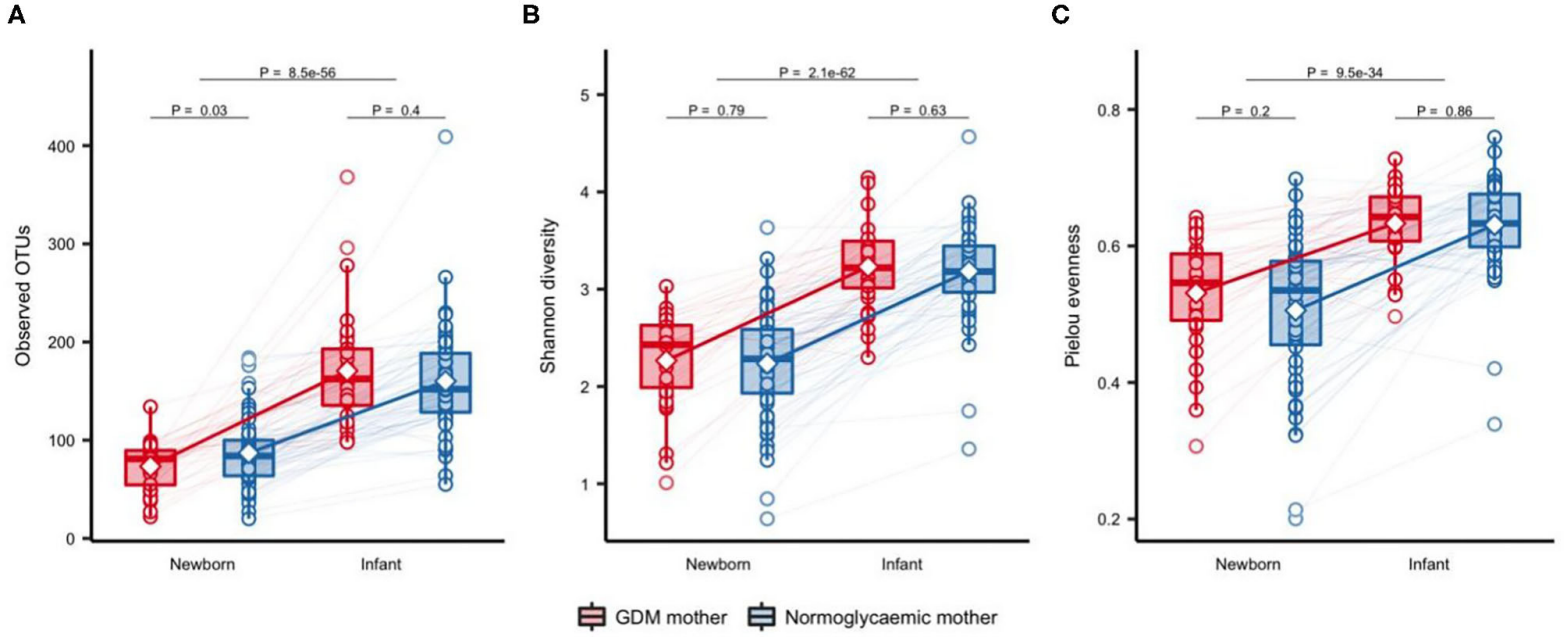
Change in alpha diversity of gut microbiota from one week of life to an average of 9 months of life. Observed OTUs **(A)**, Shannon's diversity **(B)**, and Pielou's evenness **(C)**, respectively, in offspring of mothers with GDM (*n* = 32) and mothers with normal gestational glucose regulation (*n* = 64) at one week of life and at ~9 months of life. Samples were rarefied to an equal sequencing depth of 10,000 reads. Boxes represent interquartile range (IQR), with the inside line representing the median. Whiskers represent values within 1.5×IQR of the first and third quartiles. Circles represent individual samples with lines connecting samples from the same individual. Differences between children born to mothers with GDM and children born to normoglycaemic mothers within each time point were tested using Students *t*-test. Difference between time points in all children was tested using a paired *t*-test.

Significantly fewer OTUs (*P* = 0.01) were detected in neonates born to mothers with GDM (mean = 73.9 OTUs, SD = 24.2) compared with neonates born to mothers without GDM (mean = 88.8 OTUs, SD = 35.3), with no difference in Shannon's diversity or Pielou's evenness (Figure 1). Previous studies have shown that maternal pre-pregnancy BMI, infant sex, mode of delivery and antibiotics treatment have an impact on the composition of the gut microbiota (Collado et al., 2010; Bäckhed et al., 2015; Martin et al., 2016; Hill et al., 2017). In light of this, we adjusted the analyses for these potential confounders. The difference in richness persisted after adjustment for infant sex, maternal pre-pregnancy BMI, and perinatal antibiotics exposure (Additional file 1: Supplementary Table 2), but was attenuated by adjustment for mode of delivery. Richness increased significantly during infancy (Figure 2) and at 9 months there was no difference between infants born to mothers with GDM (mean = 172.8, SD = 63.5) and infants born to mothers without GDM (mean = 156.0, SD = 60.0). Adjustment for the potential confounders mentioned above and feeding during infancy (formula supplementation vs. exclusive breastfeeding and complete vs. partial transition to solid foods) did not change the results.

### GDM Status Is Associated With Community Membership and Structure in Gut Microbiota of Offspring at First Week of Life and During Infancy

**Figure 3:**
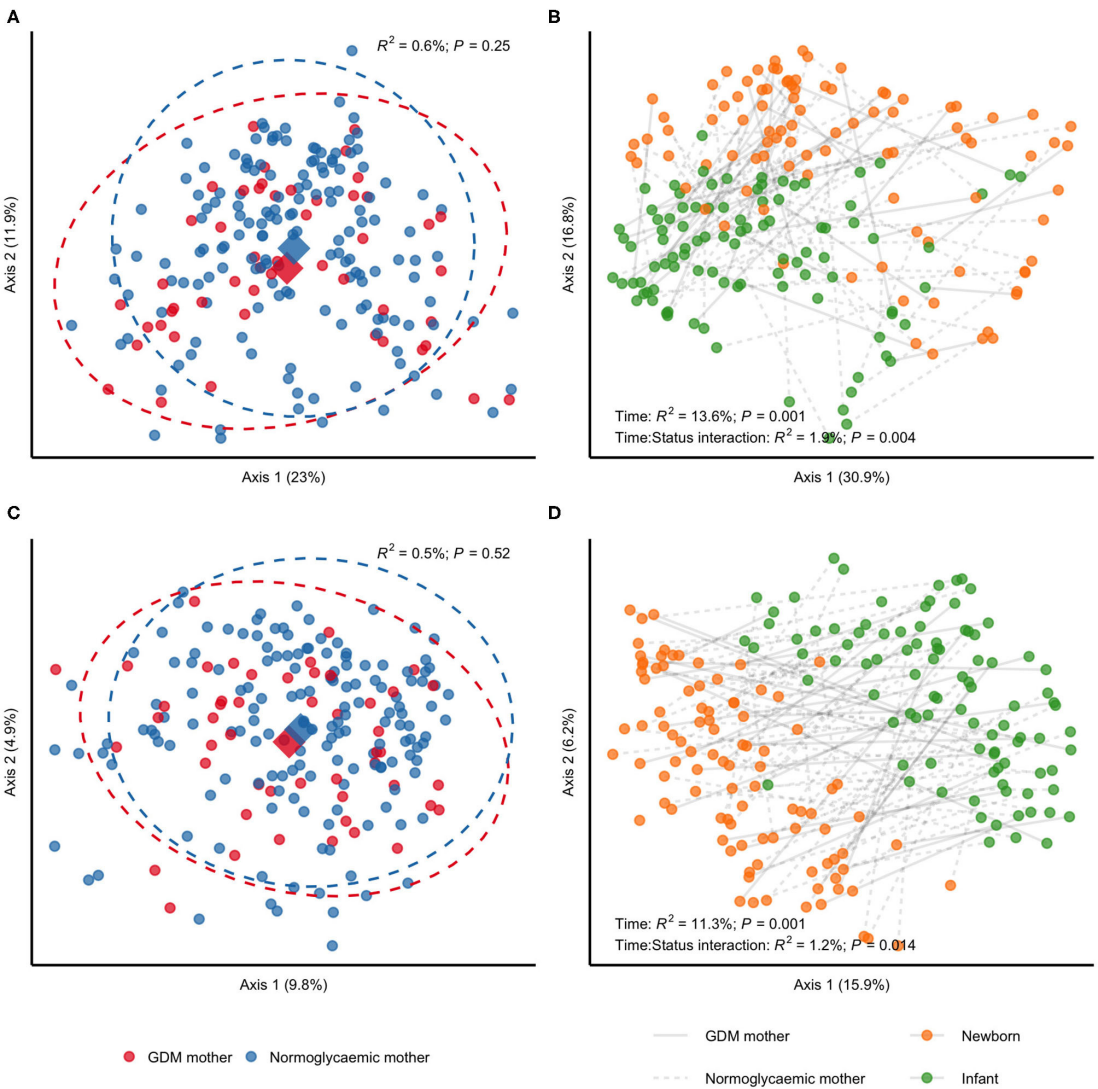
Community structure and membership of gut microbiota in children born to mothers with GDM compared with children born to mothers without GDM. All samples analyses were rarefied to an equal sequencing depth of 10,000 reads prior to principal coordinate (PCo) analysis based on weighted **(A,B)** and unweighted **(C,D)** UniFrac distances. **(A,C)** Samples from newborns of mothers with (*n* = 34) or without (*n* = 72) gestational diabetes. Points are individual samples and diamonds represent the average ordination scores and ellipses the 95% confidence intervals of a multivariate normal distribution of either group. *R*^2^ and P are from permutational multivariate analysis of variance (PERMANOVA) as implemented in the *adonis* function of the vegan R package. **(B,D)** Change in community structure from birth (1 week of life) to infancy (at an average of 9 months of life). Only samples from children examined at both time points are included (*n* = 32 and *n* = 64 for children of mothers with and without GDM, respectively). *R*^2^ and P are from PERMANOVA testing for a difference in community structure between newborn samples and samples collected during infancy (PERMANOVA model specified as (Distances ~ Time, strata = Subject), where Time is a two-level factor) and for a differential change in community structure or membership in children of mothers with GDM compared with children of mothers without GDM (PERMANOVA model specified as (Distances ~ Time:Status, strata = Subject), where Time:Status is a two-way interaction between GDM status and time with contrasts specified as GDM:Infant–GDM:Newborn–NGR:Infant + NGR:Newborn).

We detected a subtle difference in unweighted UniFrac distances when contrasting neonatal samples according to maternal GDM status in PERMANOVA analyses (*R*^2^ = 1.6%; *P* = 0.01), but no difference in weighted UniFrac (*R*^2^ = 1.5%; *P* = 0.17), indicating an effect of gestational glucose regulation on community membership. This likely reflects which bacteria are present rather than community structure that reflect abundance of bacteria (Figures 3A,C). The difference in unweighted UniFrac distances withstood adjustment for infant sex, perinatal antibiotics exposure and mode of delivery (Additional file 2: Supplementary Figure 2), and was slightly intensified by adjustment for maternal pre-pregnancy BMI (*R*^2^ = 1.9%; *P* = 0.007). From birth to infancy, we found a significant change in community structure (*R*^2^ = 13.6%; P = 0.001) and community membership (*R*^2^ = 11.3%; *P* = 0.001), represented by weighted and unweighted UniFrac distances, respectively. Longitudinally, we detected differential changes in community structure (*R*^2^ = 1.9%; *P* = 0.004) and membership (*R*^2^ = 1.2%; *P* = 0.014) from birth to infancy according to GDM status (Figures 3B,D) and the difference in community membership remained in infancy (unweighted UniFrac; *R*^2^ = 1.3%; *P* = 0.03), while community structure remained similar (unweighted UniFrac; *R*^2^ = 1.1%; *P* = 0.20). Adjustment for pre-pregnancy BMI had a minor attenuating effect on the differential change in community structure and membership, whereas adjustment for infant sex, perinatal antibiotics exposure and mode of delivery had no effect (Additional file 2: Supplementary Figure 3). Adjustment for difference in feeding during infancy had a minor attenuating effect on the differential change in community structure, but no effect on the differential change in community membership.

### Compositional Differences in Gut Microbiota of Offspring Born to Mothers With GDM

**Figure 4:**
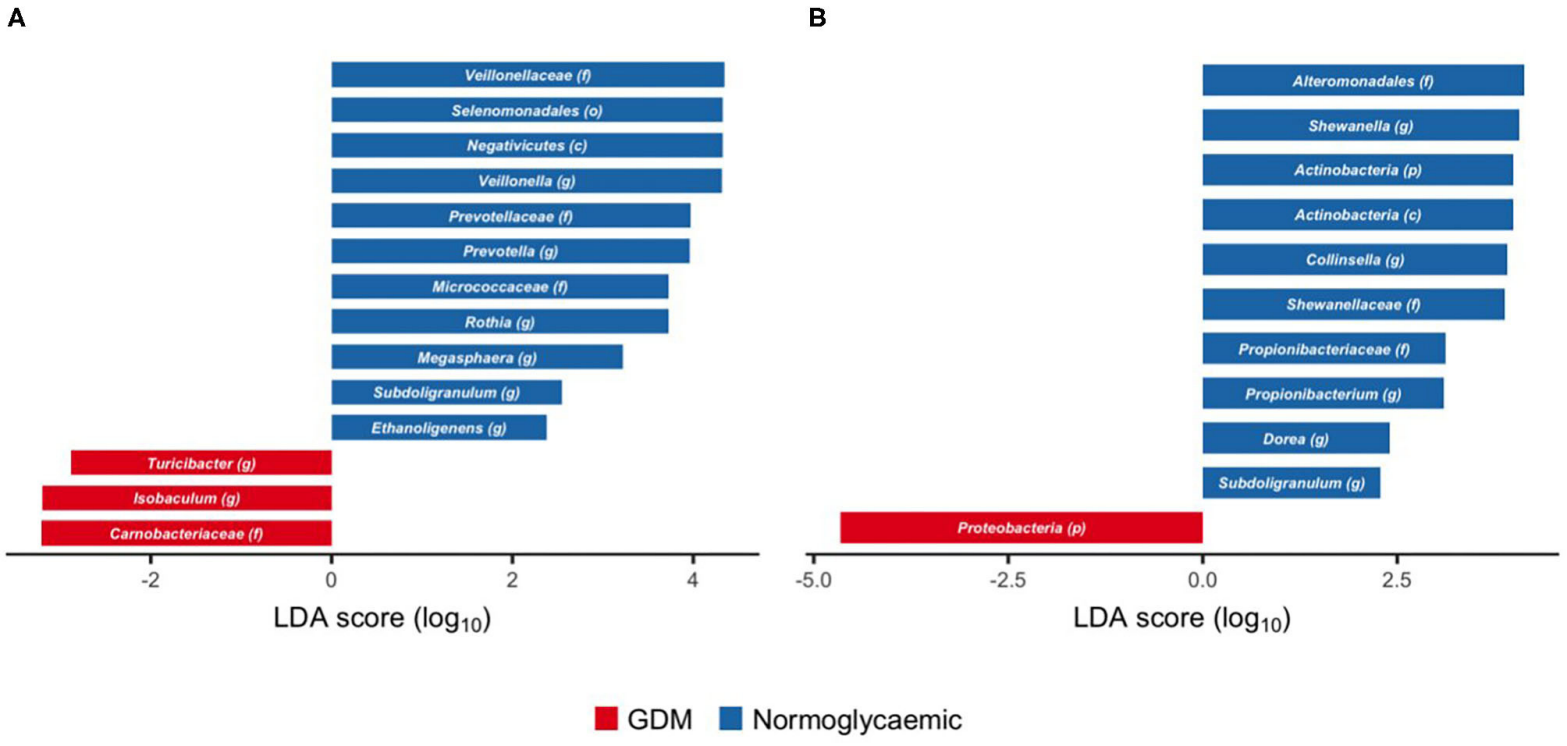
Taxonomic biomarkers of gestational diabetes in gut microbiota of newborns and infants. Scores of taxonomic biomarkers of gestational diabetes in newborns (1 week of life) **(A)** and infants (at an average of 8.8 months of life) **(B)** to genus level identified by linear discriminant analysis (LDA) using LEfSe. Color indicates the group in which a differentially abundant taxon is enriched. (p) phylum, (c) class, (o) order, (f) family, (g) genus.

Within *Firmicutes*, we identified genus *Isobaculum* and the parent family *Carnobacteriaceae* and genus *Turicibacter* as enriched in neonates born to mothers with GDM (Figure 4A), whereas the genera *Veillonella* and *Megasphaera* and their parent taxa from family to class (*Veilonellaceae, Selenomonadales*, and *Negativicutes*), as well as the genera *Subdoligranulum* and *Ethanoligenes* were depleted in neonates born to mothers with GDM. Similarly, genus *Prevotella* and the parent family *Prevotellaceae* within *Bacteroidetes*, and genus *Rothia* and the parent family *Micrococcaceae* within *Actinobacteria* were depleted in neonates born to mothers with GDM.

During infancy, phylum *Proteobacteria* was enriched in neonates born to mothers with GDM, but no subordinate taxa were associated with GDM (Figure 4B). Genus *Shewanella* and its parent family (*Shewanellaceae*) and order (*Alteromonadales*) within the same phylum were depleted in neonates born to mothers with GDM. Likewise, genus *Propionibacterium* and its parent family (*Propionibacteriaceae*) and genus *Collinsella*, both within *Actinobacteria*, were depleted in neonates born to mothers with GDM. Within *Firmicutes*, genus *Dorea*, and genus *Subdoligranulum* were depleted in neonates born to mothers with GDM.

**Figure 5:**
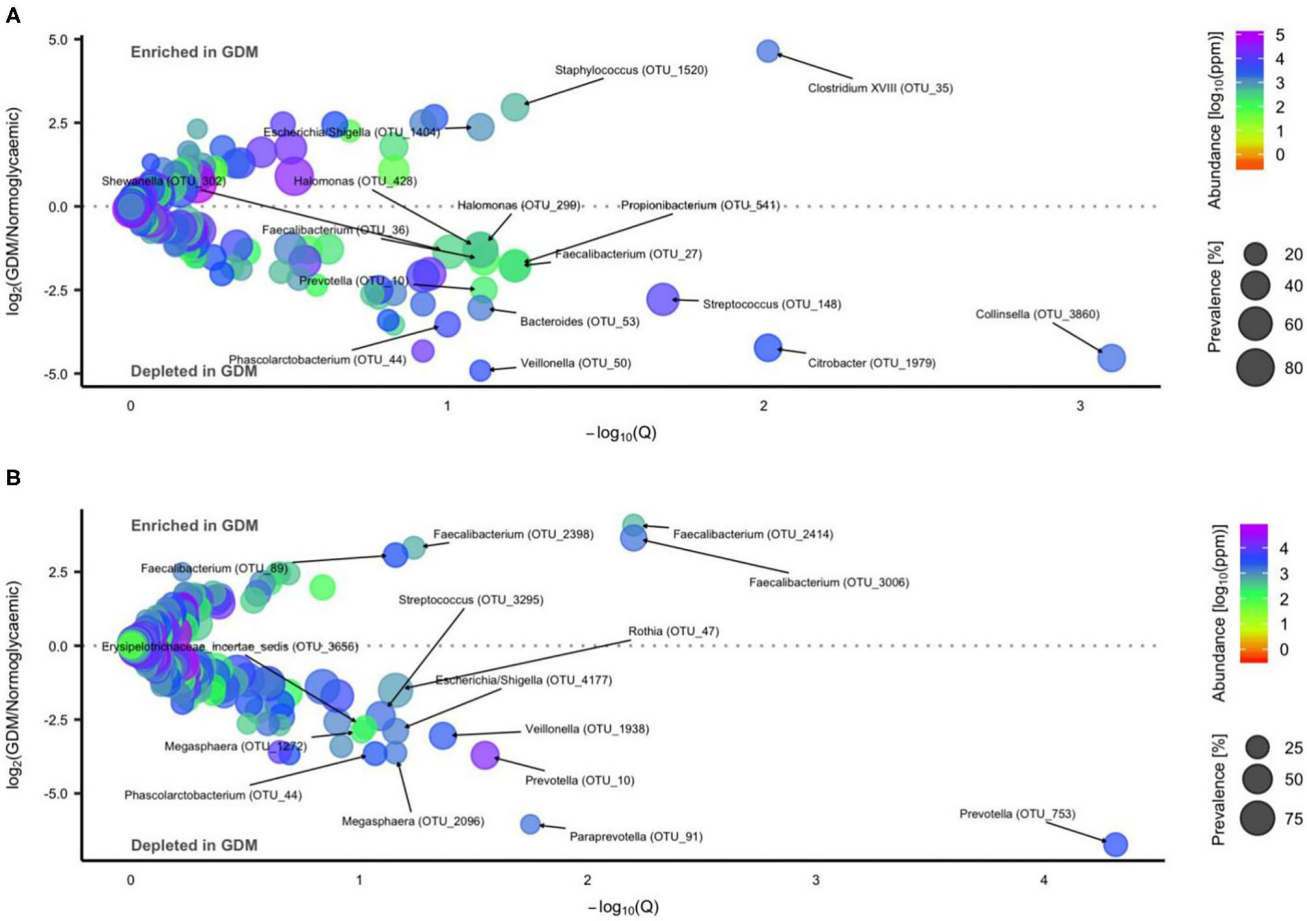
Operational Taxonomic Units (OTU's) differentially abundant in gut microbiota of newborns and infants of mothers with or without GDM. Volcano plot of estimated log_2_ fold difference in OTU abundance according to maternal GDM status in newborns [**(A)** *n* = 34 of mothers with GDM and *n* = 72 of mothers with normal gestational glucose regulation, at 1 week of life] and infants [**(B)** *n* = 42 of mothers with GDM and *n* = 81 of mothers with normal gestational glucose regulation, at an average of 8.8 months of life) and corresponding Benjamin-Hochberg adjusted P-values (Q) from negative binomial Wald tests as implemented in the DESeq2 R package. Prevalence indicates percentage of children in which a given OTU is present. Abundance indicates mean relative abundance (reads per million). Taxonomic annotation of OTUs differentially abundant at a 10% false discovery rate is given at the genus level. Detailed information is presented in Supplementary Table 3.

Using a negative binomial Wald test we identified 16 OTUs differentially abundant in neonates born to mothers with GDM or normal glucose regulation, respectively (Figure 5A, Additional file 1: Supplementary Table 3). Three OTU's were enriched while 13 OTU's including *Streptococcus, Collinsella, Phascolarctobacterium, Prevotella*, and *Faecalibacterium* were depleted in GDM neonates.

During infancy, 15 OTUs were associated with GDM status (Figure 5B, Additional file 1: Supplementary Table 3). Four OTUs were enriched in GDM infants, all assigned to *Faecalibacterium*. Eleven OTUs including *Streptococcus, Phascolarctobacterium, Veillonella*, and *Prevotella* were depleted in GDM infants.

When adjusting for mode of delivery, seven (of 16) differentially abundant OTUs in neonates and ten (of 15) during infancy remained, while revealing five new differentially abundant OTUs in neonates and six new OTUs in infants (Additional file 1: Supplementary Table 4). Adjusting for perinatal antibiotics exposure had the largest influence on the result, with only three differentially abundant OTUs remaining in neonates and eight remaining in infants (Additional file 1: Supplementary Table 5). Three new differentially abundant OTUs were revealed in infants, but none in neonates. When adjusting for infant sex, nine OTUs in neonates and four in infants remained differentially abundant according to GDM status. Twelve new differentially abundant OTUs in neonates and eight new OTUs in infants were discovered (Additional file 1: Supplementary Table 6). Adjustment for feeding during infancy (formula vs. no formula) only five abundant OTUs in infants remained, while 13 new differentially abundant OTUs were revealed (Additional file 1: Supplementary Table 7).

One *Clostridium XVIII* OTU (OTU_35) remained enriched and a *Collinsella* OTU (OTU_3860) remained depleted in neonates, when adjusted either for infant sex, mode of delivery or exposure to perinatal antibiotics. Likewise, a *Faecalibacterium* OTU (OTU_2398) remained enriched and an *Escherichia/Shigella* (OTU_4177) remained depleted in infants after adjustment either for infant sex, mode of delivery or exposure to perinatal antibiotics. However, the association of gut microbiota composition at OTU level in both neonates and infants was not statistically significant after adjustment for pre-pregnancy BMI.

### Maternal Glycaemic Traits Associated With Microbiota Diversity and Composition in Neonates and Infants

As increasing levels of maternal plasma glucose, below diagnostic threshold for GDM, have been associated with adverse pregnancy outcome (Jensen et al., 2008; The HAPO Study Cooperate Research Group, 2008) we investigated if fecal microbial diversity in offspring is associated with maternal glycaemic traits in late pregnancy. Using linear regression we found a weak negative association with maternal fasting plasma glucose and richness of the neonatal fecal microbiota (0.05% reduction in fasting plasma glucose per OTU) (Additional file 2: Supplementary Figure 4), but the association was not significant at a false discovery rate of 10%. Nor did it withstand adjustment for pre-pregnancy BMI, mode of delivery, perinatal antibiotics exposure, or newborn sex (Additional file 2: Supplementary Figures 5–8). Maternal 2-h plasma glucose, insulin sensitivity index and disposition index in late pregnancy were not associated with fecal microbial diversity in neonate samples with or without adjustments.

To identify OTUs associated with maternal fasting plasma glucose level, stimulated 2-h plasma glucose level, insulin sensitivity index, and disposition index, we divided samples into tertiles according to each of the four traits and contrasted the upper and lower tertiles. We identified 57 significant associations between 46 unique OTUs and maternal glycaemic traits in neonates without any adjustments (Additional file 1: Supplementary Table 8). Four were associated with fasting plasma glucose level, 14 associated with 2-h stimulated plasma glucose level, 15 associated with insulin sensitivity index, and 24 associated with disposition index. OTUs associated with maternal glycaemic traits in neonates were primarily belonging to phylum *Firmicutes*.

During infancy we identified 63 significant associations between 52 unique OTUs and maternal glycaemic traits (Additional file 1: Supplementary Table 9). Twenty-six associated with disposition index, 12 with insulin sensitivity index, 18 with fasting plasma glucose level and seven with 2-h stimulated plasma glucose level. Similarly to the associations in neonates, infant OTUs associated with maternal glycaemic traits were primarily belonging to phylum *Firmicutes*.

Only 14 associations with 14 unique OTUs were identified when adjusting for pre-pregnancy BMI. Nine OTUs withstood adjustment whereas five new associations appeared. Most OTUs associated with disposition index (*n* = 10), two associated with stimulated 2-h plasma glucose level and only one association with fasting plasma glucose level and insulin sensitivity index, respectively, was identified.

Several deeper resolution order taxa in neonates, including genus *Bacteroides* and all parent taxa, were associated with higher maternal disposition index with and without adjustment for pre-pregnancy BMI (Additional file 1: Supplementary Table 10). No deeper order taxa in neonates were associated with maternal fasting plasma glucose level, 2-h stimulated plasma glucose level, or insulin sensitivity index. In infants, family *Carnobacteriaceae* was associated with increased fasting plasma glucose level and lower disposition index with and without adjustment for pre-pregnancy BMI (Additional file 1: Supplementary Table 11).

### Fecal Microbiota in Offspring Associated With Gestational Weight Gain

Gestational weight gain has been found to influence the composition of the gut microbiota of the offspring (Galley et al., 2014; Stanislawski et al., 2017). We identified 19 OTUs in neonates and four OTUs in infants that associated with gestational weight gain (Additional file 1: Supplementary Table 12). Eleven OTUs in neonates were associated with lower gestational weight gain and eight OTUs were associated with higher gestational weight gain. In infants three OTUs were associated with higher gestational weight gain and only one OTU was associated with lower gestational weight gain. However, adjustment for pre-pregnancy BMI abolished all associations.

## Discussion

Our study of offspring born to mothers with GDM demonstrated that glycaemic dysregulation in late pregnancy is associated with relatively modest alterations of the gut microbiota of their neonates at first week of life and during infancy.

Children born to mothers with GDM have increased risk of developing metabolic disorders during childhood and as adults compared with children born to mothers with normal gestational glucose regulation (Clausen et al., 2008, 2009, 2013; Nehring et al., 2013; Damm et al., 2016). We found lower richness of the gut microbiota in GDM neonates compared with neonates born to mothers without GDM. This finding is in line with previous findings of both meconium and stools during first week of life in offspring of mothers with GDM compared to offspring of mothers without GDM (Su et al., 2018; Ponzo et al., 2019) and with results in adults showing a positive association between high richness and a metabolically advantageous phenotype characterized by less body-fat and lower BMI, higher insulin sensitivity, lower levels of circulating triglycerides and higher levels of HDL cholesterol, and lower levels of circulating markers of low-grade inflammation (Chatelier et al., 2013). Previous studies addressing the impact of maternal glucose dysregulation on the gut microbiota of the offspring have failed to show any association with intraindividual bacterial diversity. In a study investigating 23 term neonates, Hu et al. reported higher richness in meconium from neonates born to mothers with pre-pregnancy type 2 diabetes (*n* = 5) compared with neonates born to normoglycaemic mothers (*n* = 9). Neonates born to mothers with GDM (*n* = 5) had similar richness compared with neonates born to normoglycaemic mothers (Hu et al., 2013). The lack of difference in richness in the study by Hu et al. might be due to the relatively few neonates born to mothers with GDM. Timing of sample collection may also be of importance. At 1 month of age, no differences in alpha-diversity between 15 infants born to mothers with GDM compared with 76 infants born after a glucose tolerant pregnancy were reported (Koren et al., 2012). Our results suggest that GDM offspring are born with lower gut microbiota richness, but catch up during infancy and have similar gut bacterial richness as offspring born to normoglycaemic mothers at 9 months of age. The inconsistency of findings in the literature with our findings might partially be related to differences in feeding approaches including length of breastfeeding, formula feeding and time for introduction of solid food.

We found several OTUs and deeper resolution order taxa associated with GDM offspring at first week of life and during infancy. In contrast, a previous study did not find any OTUs associated with GDM status at 1 month of life of infants, possibly due to a small sample size of infants born to mothers with GDM (*n* = 15) (Koren et al., 2012). At first week of life we found OTUs primarily belonging to *Firmicutes* and *Proteobacteria*, depleted in neonates born to mothers with GDM. Along this finding it has been reported that *Proteobacteria* were depleted in meconium of neonates born to mothers with pre-pregnancy type 2 diabetes (Fugmann et al., 2015). However, the abundance of *Proteobacteria* in newborns have previously been associated with maternal GDM (Su et al., 2018), which might represent different strains of *Proteobacteria* as well as different sequencing methods.

Our findings in newborns of mothers with GDM show similarities with the gut microbiota in children with overweight and adults with metabolic disorders (Kalliomäki et al., 2008; Zhang et al., 2013; Moreno-Indias et al., 2016). We found a *Staphylococcus* species which were enriched in neonates born to mothers with GDM. Increased abundance of *Staphylococcus aureus* during infancy has previously been associated with overweight at 7 years of age (Kalliomäki et al., 2008). Furthermore, one *Streptococcus* OTU was depleted in infants born to mothers with GDM and associated with higher maternal disposition index. This is in line with findings in in adults, where the abundance of *Streptococcus* is depleted in individuals with pre-diabetes and type 2 diabetes (Zhang et al., 2013). Interestingly, a *Pharscolarctobacterium* species was depleted in neonates of mothers with GDM during first week of life and in infancy. Depletion of *Pharscolarctobacterium* has been reported in adults with insulin resistance (Moreno-Indias et al., 2016) and in patients with ulcerative colitis and Crohn's disease (Guinane and Cotter, 2013), suggesting a potentially anti-inflammatory effect of this species.

In neonates, the genus *Prevotella* was a taxonomic biomarker of normal gestational glucose regulation. One *Prevotella* OTU, which was depleted in neonates and infants born to mothers with GDM, was in neonates associated with lower maternal stimulated plasma glucose level, and was in infants associated with lower maternal fasting plasma glucose level, higher disposition index, and higher insulin sensitivity. Previous studies have shown *Prevotella* to be depleted in children with type 1 diabetes (Murri et al., 2013), and in lower abundance in meconium of newborns born to mothers with GDM compared with mothers without GDM (Su et al., 2018), while certain *Prevotella* species i.e., *Prevotella copri* are associated with insulin resistance in adults and are able to induce the same phenotype in high-fed mice (Pedersen et al., 2016). These findings indicate that *Prevotella* is a heterogeneous clade, with different strains having either deleterious or favorable effects on host metabolism; effects which are likely contextual, e.g., dependent on host dietary habits. Disentangling these effects of *Prevotella* require deeper taxonomic resolution than 16S amplicon sequencing can provide.

Species within *Faecalibacterium* displayed different patterns during first week of life and in infancy. In neonates, two *Faecalibacterium* species were depleted in newborns of mothers with GDM and associated with lower maternal fasting plasma glucose level, whereas in infants four other *Faecalibacterium* species were enriched in children born to mothers with GDM. Interestingly, we found another OTU of *Faecalibacterium* enriched in infants to mothers with GDM after adjusting for infant sex and perinatal antibiotic exposure. When comparing the gut microbiota of children in our study with the gut microbiota composition of their mothers we found the same OTU of *Faecalibacterium* enriched in mothers with GDM both in late pregnancy and 9 months postpartum (the same time point as our samples from infants were collected) (Crusell et al., 2018). In non-pregnant adults with metabolic syndrome and type 2 diabetes *F. prausnitzii* is depleted (Zhang et al., 2013; Haro et al., 2016), but in the childhood *Faecalibacterium* is reported to correlate positively with BMI (Riva et al., 2017). In children the relationship between *Faecalibacterium* and metabolic health appears more complex, than in adults, where *Faecalibacterium* as a dominant butyrate producing taxa (Louis and Flint, 2009) has been consistently associated with a healthier metabolic phenotype.

Several of the OTUs which were depleted in neonates born to mothers with GDM were also associated with maternal glycaemic traits in late pregnancy. Genus *Collinsella* was a biomarker of normal gestational glucose regulation in infants. Intriguingly, a *Collinsella* OTU was depleted in infants born to mothers with GDM after adjustment for infant sex, mode of delivery, and perinatal antibiotics exposure and this OTU associated with higher maternal disposition index during first week of life and in infancy. During infancy, the same *Collinsella* OTU also associated with lower maternal fasting plasma glucose. Interestingly, when comparing the gut microbiota composition in our study group of children to the gut microbiota composition in their mothers at the same time point as the stool collection for the infants, we found that the same OTU of *Collinsella* was depleted in the women with previous GDM (Crusell et al., 2018). *Collinsella* has previously been reported to be enriched in adults with type 2 diabetes and high abundance of *Collinsella* in infants during the first 6 month of age has been associated with adiposity in toddlers (Dogra et al., 2015; Lambeth et al., 2015). With the previous knowledge of species belonging to the genus *Collinsella* we would have expected enrichment of the species in the children born to mothers with GDM, as they have increased risk of development of metabolic disorders and obesity later in life (Clausen et al., 2009; Nehring et al., 2013). However, in our study *Collinsella* species seem to associate with a healthier metabolic profile and perhaps a beneficial effect for the infants. The contrasting findings might be due to the applied extraction method, amplicon primers and sequencing methods chosen in the studies. It might also be that various strains of *Collinsella* associate with different metabolic profiles of the host.

We showed that adjustment for mode of delivery, perinatal antibiotics exposure and infant sex influenced our results. This is in line with previous reports (Collado et al., 2010; Bäckhed et al., 2015; Martin et al., 2016; Hill et al., 2017) and emphasize that the gut microbial community, even in neonates, might be influenced by numerous factors. Mode of delivery could influence the first colonizing microbes of the gut and thereby also the development of microbiota composition in infants similarly to perinatal antibiotics exposure. However, when we adjusted for these factors, associations between maternal GDM status and gut microbiota composition in the children were still present, suggesting that maternal metabolic health during pregnancy is important for gut composition development in offspring.

Gut microbiota composition in infants has also as discussed been reported to associate with feeding patterns (Bäckhed et al., 2015; Hill et al., 2017) as well as time for cessation of breastfeeding (Koenig et al., 2011; Bergström et al., 2014; Bäckhed et al., 2015). We did not see a substantial effect when adjusting for feeding (formula supplementation vs. exclusive breastfeeding and complete vs. partial transition to solid foods). The lack of effect of feeding could be attributable to the crudeness of the available dietary data, which do not take into account the timing of feeding milestones (e.g., introduction of supplementary formula and introduction of solid foods). However, the lack of effect might be also be due to the uniformity of the data. When sampled during the first week of life, all neonates were breast fed. Only three neonates were breastfed <1 month and when sampled in infancy all children had been introduced to solid foods, with approximately half of the infants still being breastfed and with no differences between the two groups of infants.

A limitation of our study is that we do not have information about the exact time for introduction of solid food in the infants and therefore we do not know for how long the infants have been eating solid food. However, all infants had been introduced to solid food when their samples in infancy were collected. Another limitation of the project is the range of age between the infants at the time of examinations. The first examination and stool samples during the neonate period was between 2 and 7 days of life, which we find is an acceptable range. However, at the time of follow-up there was a larger range [6–18 months, however most of the infants were 8–9 months old (25 and 75th percentile)] of age due to difficulties with getting the participants to attend the examinations. Furthermore, our study is based on a convenience sample from a previous research project (Crusell et al., 2018) where the two groups of mothers were congruent in regards to presence of risk factors for developing GDM during pregnancy. Very few women (*n* = 9) had GDM according to the criteria used in clinical practice in Denmark. Consequently, lifestyle interventions i.e., change in diet or physical activity are unlikely to affect our results. One could speculate, that the differences we observe in the gut microbiota of women for whom the gestational hyperglycemia is left to run its course are attenuated by lifestyle or pharmacologic intervention. In the present study, however, most cases of GDM were identified by a relatively mild elevation in fasting plasma glucose. Further studies are needed to examine whether lifestyle interventions in late pregnancy has any effect on the gut microbiota of the offspring, especially in women with isolated mild fasting hyperglycemia.

*Bacteroidetes* and *Bifidobacteria* have been reported as first colonizers of infant guts in both human and murine studies (Pantoja-Feliciano et al., 2013; Jost et al., 2014; Mueller et al., 2015). One study, however, found that *Bifidobacteria* only occur in a fraction of infants and are not numerically dominant (Palmer et al., 2007). A limitation of our study was that *Bifidobacteria* was low abundant in all our samples, likely because the primers used were suboptimal for amplification of *Bifidobacteria* (Walker et al., 2015), limiting our insights into this health-related branch of the phylogenetic tree. Based on the results of fecal microbiota composition at 1 week and 9 months of life it is difficult to determine if some species are healthier than others in relations to the long-term metabolic risk for offspring of mothers with GDM. Gut microbiota are known to develop throughout childhood and numerous factors can influence this development (Penders et al., 2006; Azad et al., 2013; Levin et al., 2016). Still, in the present study we could demonstrate differences at first week of life and in infancy between children born to mothers with and without GDM. Our findings might suggest that some bacterial species could be beneficial or even protective as they are depleted both during first week of life and in infancy, while enrichment of other species might be disadvantageous in regards to future metabolic health. We furthermore found similarities in gut microbiota composition between the mothers with GDM and their children. We therefore hypothesize that mothers with GDM, by vertical transmission of microbes, make their offspring more prone to develop metabolic diseases later in life. To address this hypothesis, prospective studies are obviously needed where the same individuals are followed at various stages of life.

## Conclusion

We have explored the gut microbiota of neonates and of infants at 9 months of life in relation to the glucose regulation of their mothers. Intriguingly, the relatively modestly altered taxonomic composition in offspring of mothers with GDM has similarities with the gut microbiota composition found in childhood obesity and in adults with type 2 diabetes. Since children born after a gestational diabetic pregnancy have increased risk of developing overweight and other metabolic abnormalities we hypothesize that this proneness in part may be caused by an aberrant gut microbiota in early life. Our study design did, however, not allow us to explore this hypothesis. To test the idea that vertical transmission of intestinal microbes is a risk factor for development of metabolic disorders in the offspring, carefully designed longitudinal investigations through childhood, adolescence, and adulthood are needed. Such efforts might also include studies of maternal gut microbiota composition and function during pre-conceptive period, pregnancy, as well as gestational-, perinatal-, and early postnatal factors influencing gut microbiota of the mother and the offspring.

## Data Availability Statement

16S RNA sequencing data has been uploaded to ENA; accession number: PRJEB40425200917.

## Ethics Statement

The studies involving human participants were reviewed and approved by the Ethical Committees of the Capital Region of Denmark (Protocol #H-4-2013-10). Written informed consent to participate in this study was provided by the participants' legal guardian/next of kin.

## Author Contributions

MC conceived and conducted the study, performed analyses, the interpretation of results and the drafting of the manuscript. THH performed the analyses and interpretations of the result and drafting of the manuscript. TN and KA assisted with the interpretation of the results and drafting of the manuscript. JL and PD conceived the study and assisted in drafting the manuscript. F-AH handled the fecal sample in regards to amplification and sequencing. MR performed the sequencing and quality control of data. AF, HV, NJ, CR, OC, and TH assisted in drafting of the manuscript. OP conceived and supervised all phases of the study and assisted with the interpretation of the results and drafting of the manuscript. All authors have read and approved the final manuscript.

## Conflict of Interest

The authors declare that the research was conducted in the absence of any commercial or financial relationships that could be construed as a potential conflict of interest.

## Abbreviations

BMI: Body Mass Index
DNA: Deoxyribonucleic acid
GDM: gestational diabetes mellitus
HOMA-IR: Homeostasis model assessment of insulin resistance
OGTT: Oral Glucose Tolerance Test
OUT: Operational Taxonomic Unit
rRNA: ribosomal RNA gene.
