# Supplementary material for: Comparative Studies of the Gut Microbiota in the Offspring of Mothers With and Without Gestational Diabetes
Source: Front Cell Infect Microbiol. 2020 Oct 23;10:536282. doi: 10.3389/fcimb.2020.536282 (PMC7645212; doi:10.3389/fcimb.2020.536282)
Supplement: Supplementary file 2 [file Data_Sheet_2.doc]

**Supplementary figures**

**Figure S1. Flowchart**


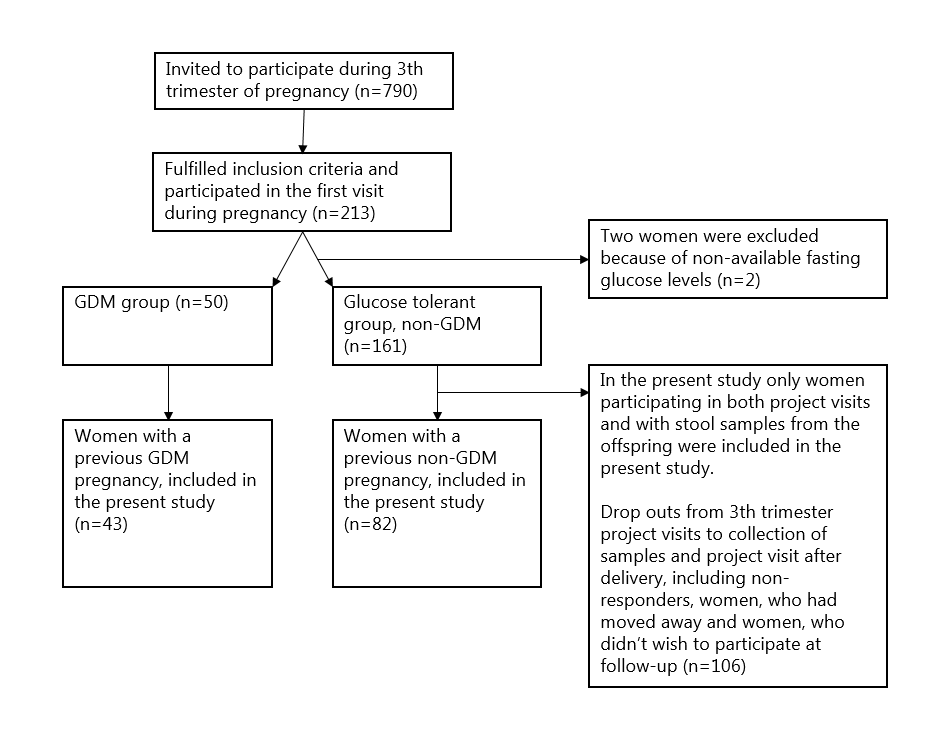


790 women were invited to participate in the project as they fulfilled the inclusion criteria. Two hundred and thirteen women accepted and participated in the first project visit during third trimester. Two women were excluded as fasting plasma glucose was not available. The included women were grouped according to the result of the oral glucose tolerant test (OGTT) in a glucose tolerant group (n=161) and a gestational diabetes mellitus (GDM) group (n=50). All participants were invited for a second project visit after delivery, 125 accepted; 82 women had had a previous normoglycaemic pregnancy and 43 women had had a previous GDM pregnancy. Eighty six women did not want to participate in the rest of the project for personal reasons or had moved. Twenty women did not respond to the invitation to project visit after delivery. There were no significant differences in phenotype characteristics between the women, who completed the second project visit compared to the drop-outs.

**Figure S2. Community structure and membership in gut microbiota of newborns to mothers with and without GDM; adjustment for covariates.**


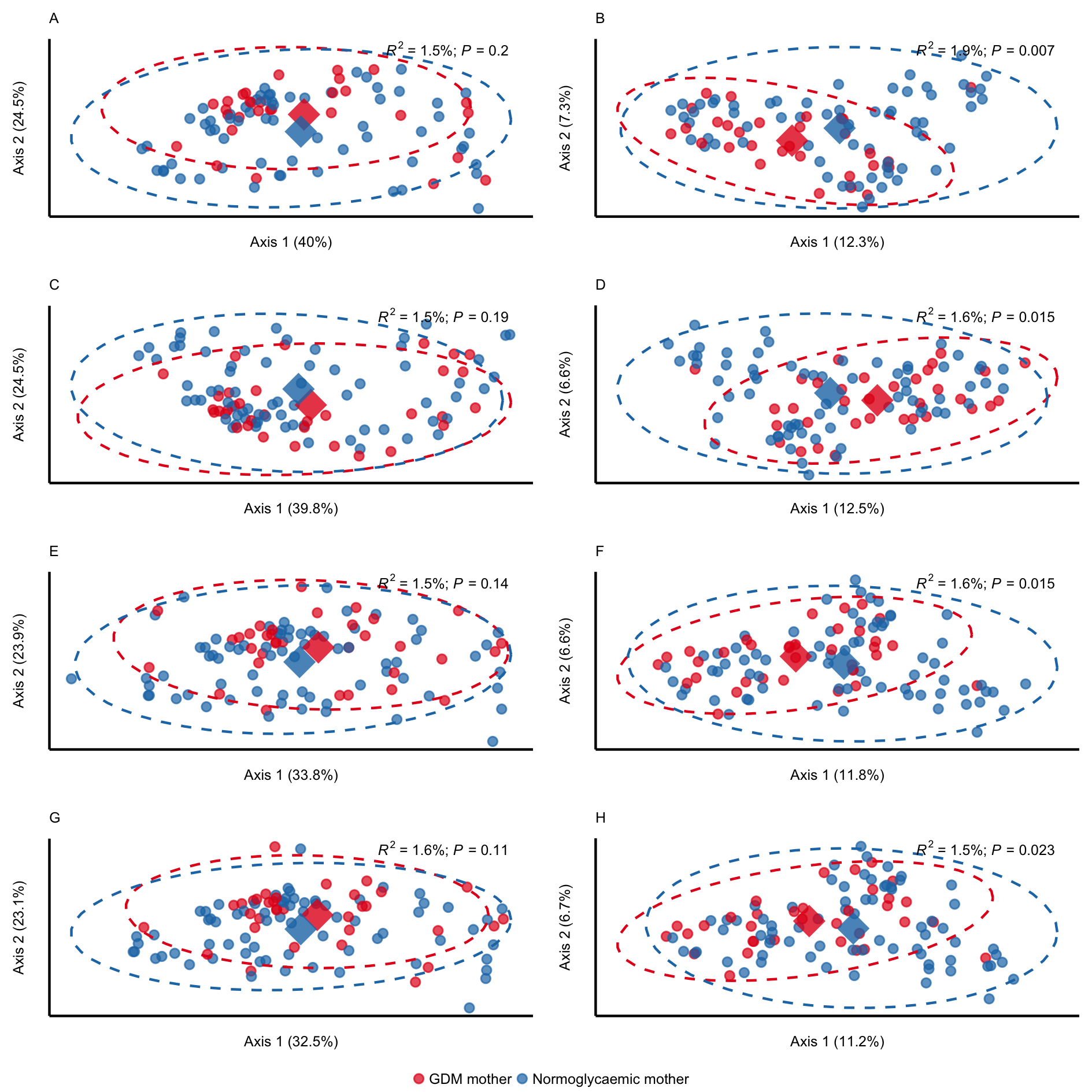


Principal coordinate ordination of weighted [A,C,E,G] and unweighted UniFrac distances [B,D,F,H], adjusted for pre-pregnancy BMI [A,B], infant sex [C,D], perinatal antibiotics [E,F] and delivery mode [G,H]. R^2^ and P are from permutational multivariate analysis of variance contrasting newborns born to mothers with GDM and newborns born to mothers with normal gestational glucose regulation.

**Figure S3. Longitudinally changes in community structure of gut microbiota in newborns and infants born to mothers with and without GDM; adjustment for covariates.**


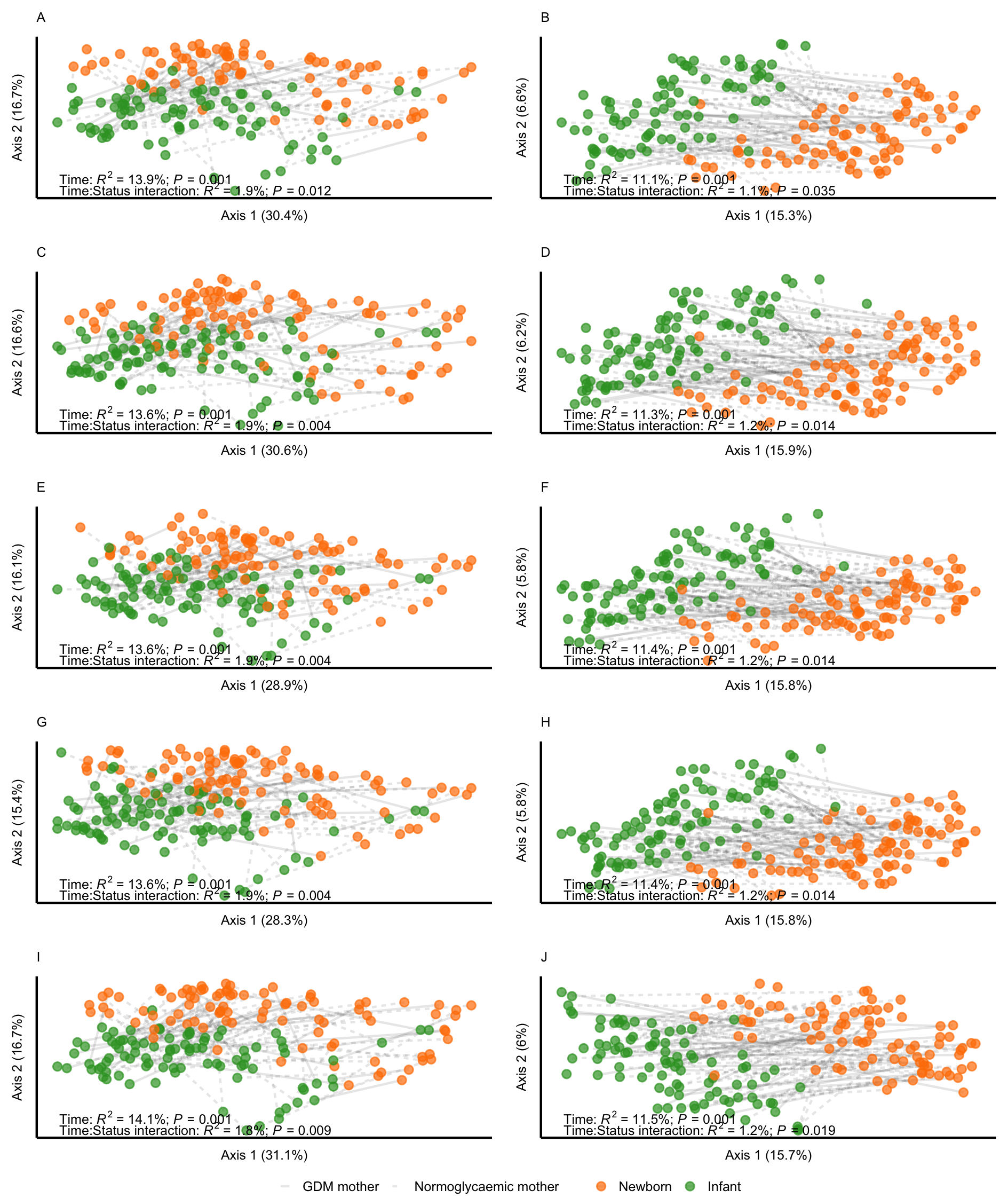


Principal coordinate ordination of weighted [A,C,E,G] and unweighted UniFrac distances [B,D,F,H] adjusted for pre-pregnancy BMI [A,B], infant sex [C,D], perinatal antibiotics [E,F] and delivery mode [G,H]. R^2^ and P are from PERMANOVA testing for a difference in community structure between newborn samples (one week of life) and samples collected during infancy (at an average at 8.8 months of life), and for a differential change in community structure in children born to mothers with GDM compared with children born to mothers without GDM.

**Figure S4. Relationship between maternal glycaemic traits and estimates of alpha diversity of gut microbiota in newborns according to maternal GDM status**


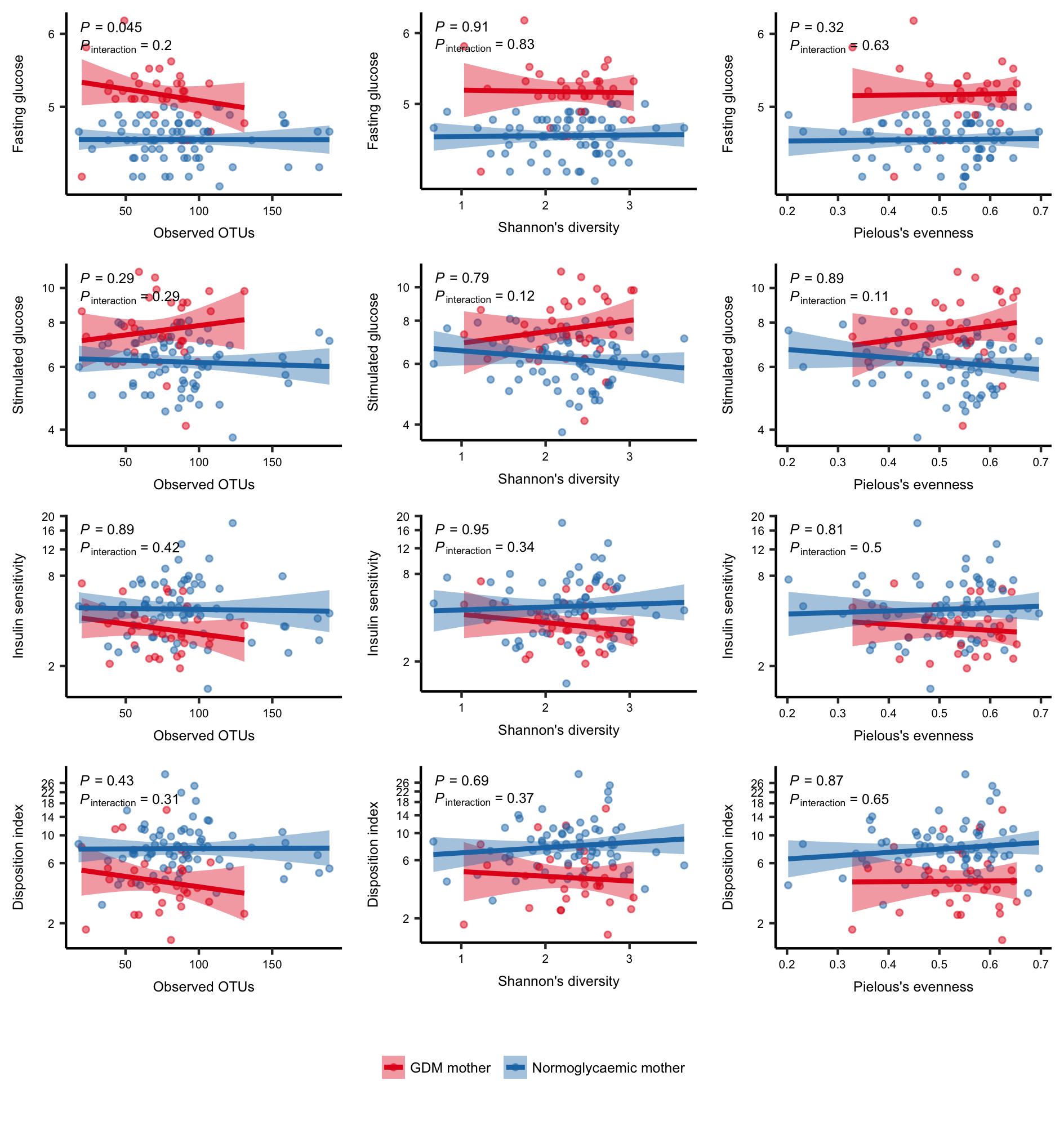


Scatter plots showing the relationships between four maternal glycaemic traits (fasting and 2 hour stimulated plasma glucose, insulin sensitivity index and disposition index; 10_10_ scaled) in third trimester of pregnancy and three measures of neonatal alpha diversity (observed OTUs, Shannon’s diversity index and Pielou’s evenness index). Regression lines with 95% confidence intervals are plotted for newborns born to mothers with GDM (red) and newborns born to normoglycaemic mothers (blue) individually. *P* indicate the nominal significance of the linear relationship between each glycaemic trait and alpha diversity measure (linear regression) in newborns born to mothers with GDM and newborns born to normoglycaemic mothers combined. P_interaction_ indicate the nominal significance of the interaction between alpha diversity and GDM status for each combination of alpha diversity measure and glycaemic trait.

**Figure S5. Relationship between maternal glycaemic traits and estimates of alpha diversity of gut microbiota in newborns according to maternal GDM status; adjustment for maternal pre-pregnancy BMI**


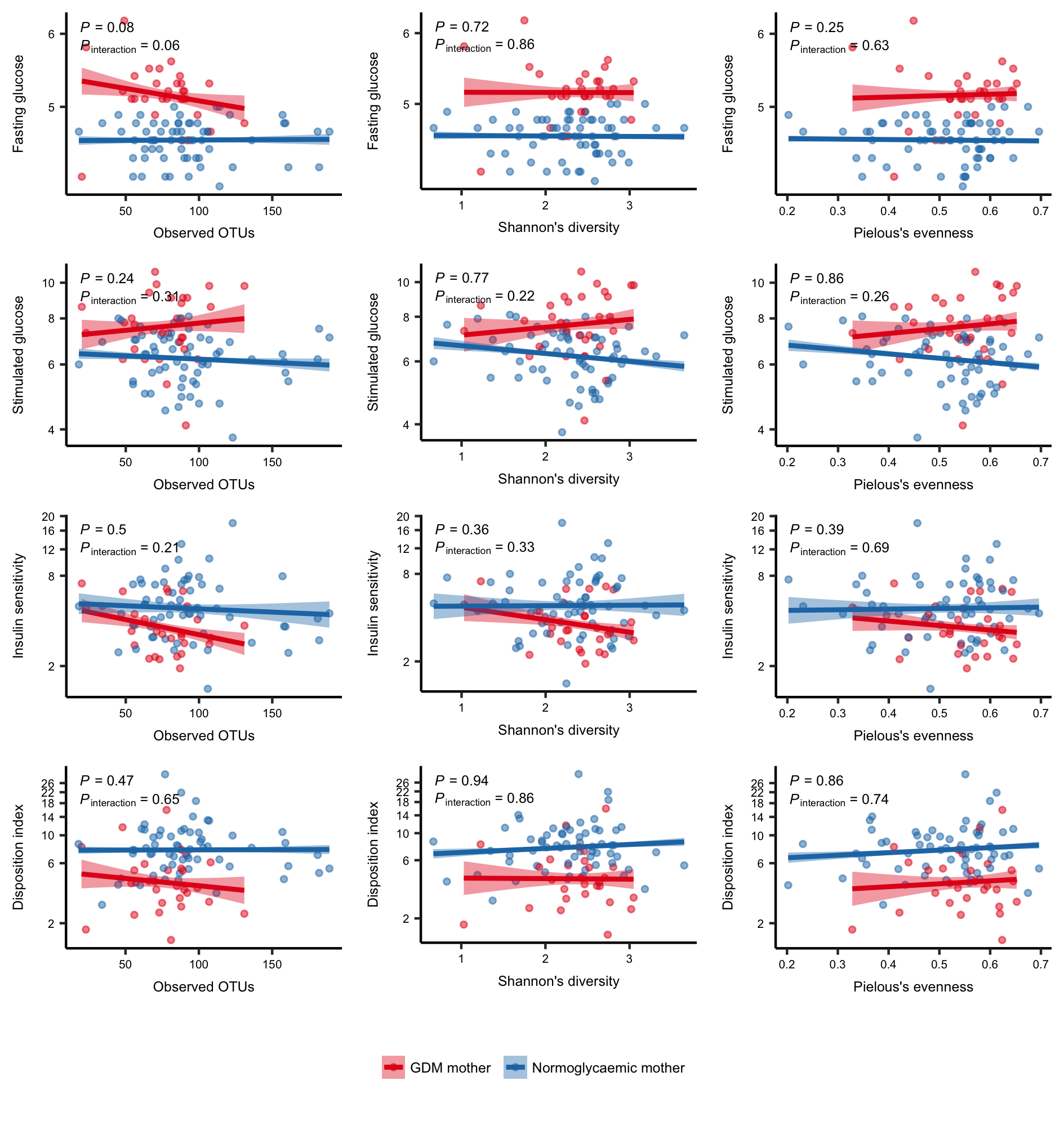


Scatter plots showing the relationships between four maternal glycaemic traits (fasting and 2 hour stimulated plasma glucose, insulin sensitivity index and disposition index; 10_10_ scaled) in third trimester of pregnancy and three measures of alpha diversity (observed OTUs, Shannon’s diversity index and Pielou’s evenness index) adjusted for maternal pre-pregnancy BMI. Regression lines with 95% confidence intervals are plotted for newborns born to mothers with GDM (red) and newborns born to normoglycaemic mothers (blue) individually. *P* indicate the nominal significance of the linear relationship between each glycaemic trait and alpha diversity measure (linear regression) in newborns born to mothers with GDM and newborns born to normoglycaemic mothers combined. P_interaction_ indicate the nominal significance of the interaction between alpha diversity and GDM status for each combination of alpha diversity measure and glycaemic trait.

**Figure S6. Relationship between maternal glycaemic traits and estimates of alpha diversity of gut microbiota in newborns according to maternal GDM status; adjustment for mode of delivery**


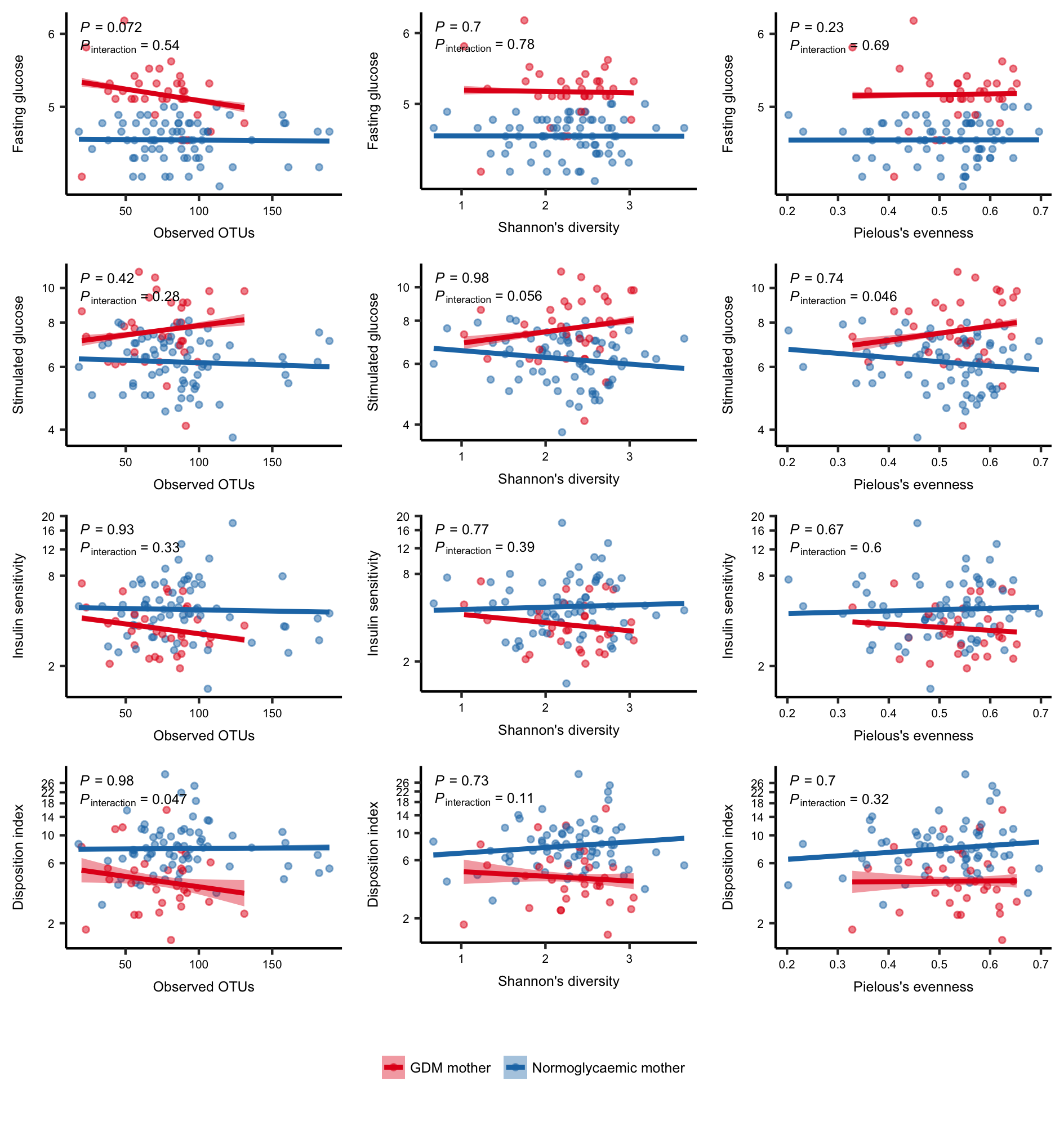


Scatter plots showing the relationships between four maternal glycaemic traits (fasting and 2 hour stimulated plasma glucose, insulin sensitivity index and disposition index; 10_10_ scaled) in third trimester of pregnancy and three measures of alpha diversity (observed OTUs, Shannon’s diversity index and Pielou’s evenness index) with adjustment for delivery mode. Regression lines with 95% confidence intervals are plotted for newborns born to mothers with GDM (red) and newborns born to normoglycaemic mothers (blue) individually. *P* indicate the nominal significance of the linear relationship between each glycaemic trait and alpha diversity measure (linear regression) in newborns born to mothers with GDM and newborns born to normoglycaemic mothers combined. P_interaction_ indicate the nominal significance of the interaction between alpha diversity and GDM status for each combination of alpha diversity measure and glycaemic trait.

**Figure S7. Relationship between maternal glycaemic traits and estimates of alpha diversity of gut microbiota in newborns according to maternal GDM status; adjustment for perinatal antibiotics exposure**


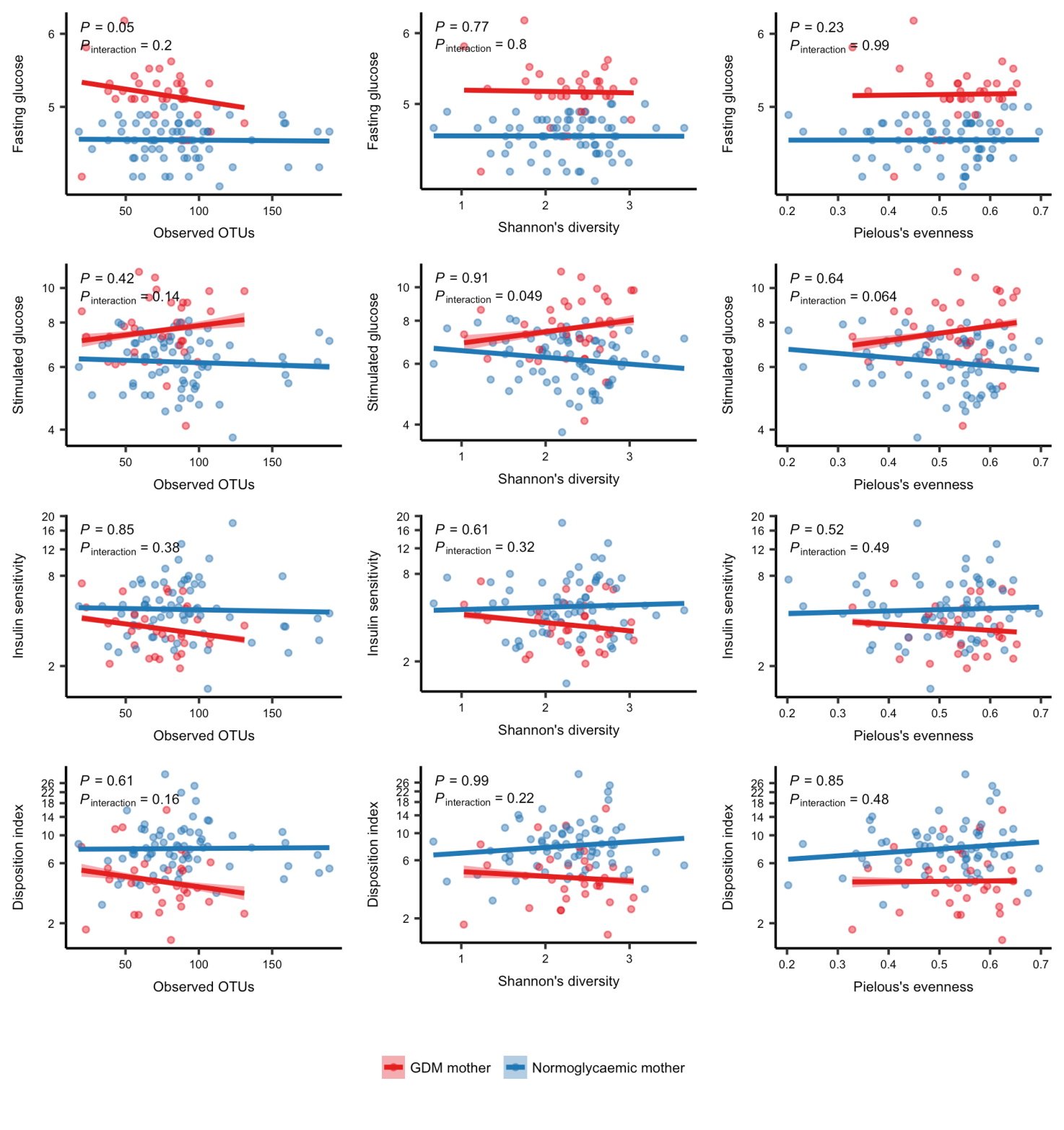


Scatter plots showing the relationships between four maternal glycaemic traits (fasting and 2 hour stimulated plasma glucose, insulin sensitivity index and disposition index; 10_10_ scaled) in third trimester of pregnancy and three measures of alpha diversity (observed OTUs, Shannon’s diversity index and Pielou’s evenness index) with adjustment for perinatal antibiotics exposure. Regression lines with 95% confidence intervals are plotted for newborns born to mothers with GDM (red) and newborns born to normoglycaemic mothers (blue) individually. *P* indicate the nominal significance of the linear relationship between each glycaemic trait and alpha diversity measure (linear regression) in newborns born to mothers with GDM and newborns born to normoglycaemic mothers combined. P_interaction_ indicate the nominal significance of the interaction between alpha diversity and GDM status for each combination of alpha diversity measure and glycaemic trait.

**Figure S8. Relationship between maternal glycaemic traits and estimates of alpha diversity of gut microbiota in newborns according to maternal GDM status; adjustment for sex of newborns**


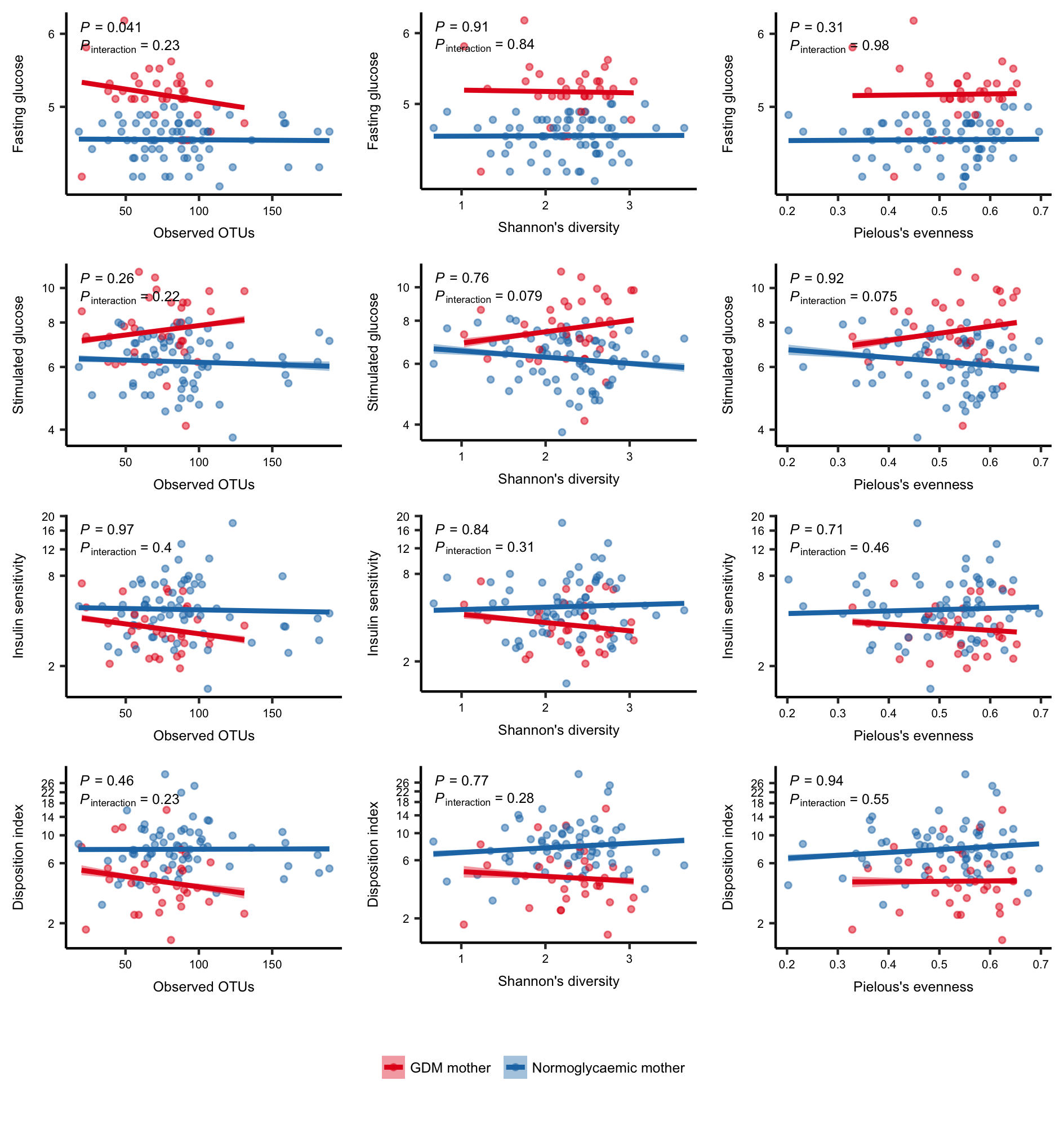


Scatter plots showing the relationships between four maternal glycaemic traits (fasting and 2 hour stimulated plasma glucose, insulin sensitivity index and disposition index; 10_10_ scaled) in third trimester of pregnancy and three measures of alpha diversity (observed OTUs, Shannon’s diversity index and Pielou’s evenness index) with adjustment for sex of newborns. Regression lines with 95% confidence intervals are plotted for newborns born to mothers with GDM (red) and newborns born to normoglycaemic mothers (blue) individually. *P* indicate the nominal significance of the linear relationship between each glycaemic trait and alpha diversity measure (linear regression) in newborns born to mothers with GDM and newborns born to normoglycaemic mothers combined. P_interaction_ indicate the nominal significance of the interaction between alpha diversity and GDM status for each combination of alpha diversity measure and glycaemic trait.
